# The Impact of Wildflower Habitat on Insect Functional Group Abundance in Turfgrass Systems

**DOI:** 10.3390/insects15070520

**Published:** 2024-07-11

**Authors:** Laura E. Hamon, Lauren D. Kilpatrick, Terri L. Billeisen

**Affiliations:** 1 Department of Entomology & Plant Pathology, North Carolina State University, Raleigh, NC 27695, USA; tlhoctor@ncsu.edu; 2 Department of Horticulture, North Carolina State University, Raleigh, NC 27695, USA; laurenkilpatrick27@gmail.com

**Keywords:** turfgrass, arthropod conservation, habitat management, urban ecology, pollinators, natural enemies

## Abstract

**Simple Summary:**

Turfgrass is a prevalent ground cover type within urban areas, and is found on large swathes of commercial, residential, and recreational land. Turfgrass supports low biodiversity compared with more diverse vegetation communities. One increasingly popular method for supporting arthropod communities in urban areas is to plant wildflowers in available spaces using a commercial seed mix, which can support beneficial insects such as butterflies, bees, and natural enemies. However, the long-term impacts of these wildflower habitats on arthropod communities in turfgrass systems is poorly known. To explore this, we used pan traps to sample insects from turfgrass systems adjacent to wildflower plots over three years and quantified how different insect groups changed in abundance. We found that different insect groups had variable changes in abundance over time, with sweat bees (Halicitdae) and skipper butterflies (Hesperiidae) being less abundant two years after wildlife implementation, and predatory flies showing boosted abundance for two years post-implementation. These results point to the complex dynamics of arthropod communities in turfgrass systems following wildflower implementation and the need to study how to best maintain these habitats to support long-term biodiversity.

**Abstract:**

Urbanization is rapidly influencing the abundance and diversity of arthropods. Within urban systems, managed turfgrass is a prominent land cover which can support only a limited number of arthropod groups. To allow for more arthropod biodiversity and to support beneficial insects within turfgrass, increasing numbers of land managers are choosing to partially convert turf habitat to wildflower habitat using commercially available seed mixes. However, the population dynamics of arthropod groups in these systems are poorly known, with consequentially little information on best long-term practices for managing wildflower habitats in turfgrass systems. To address this gap, we sampled insects using pan traps in turfgrass systems pre- and post-implementation of wildflower habitats and examined the change in abundance of several insect families and functional guilds. Insect groups had variable responses to wildflower habitat implementation, with some groups such as sweat bees and skipper butterflies showing a decline two years post-implementation. Other groups, such as predatory flies, were relatively more abundant one and two years post-implementation. These variable responses point to the need for more research on the long-term effects of wildflower habitats on beneficial insects in turfgrass habitats.

## 1. Introduction

Rapid urbanization in the USA has been widely documented, with over 890,000 hectares of land converted to urban area each year [1], and it has been implicated in lower abundance of arthropods and reduced species richness in many regions globally [2,3]. Turfgrass is a prevalent plant system in urban areas, particularly in residential, commercial, and recreational spaces. Although turfgrass supports higher arthropod abundance and diversity compared with several other urban ground cover types such as bare ground and wood chips [4,5,6], managed turfgrass supports lower arthropod biodiversity compared with more diverse and structurally complex plant communities [7]. Highly managed turfgrass systems also experience frequent disturbance and chemical inputs in the form of mowing and pesticides, further limiting their ability to support diverse arthropod communities [8,9,10]. To offset this trend, it is essential to investigate the potential methods by which green spaces can potentially support and augment arthropod populations.

One increasingly popular method for supporting arthropods in urban areas is planting wildflower habitats. Wildflower habitats provide pollen and nectar resources, alternative prey, and refuge from frequent disturbance [11,12,13]. Adding wildflower habitats to turfgrass areas such as golf courses can support elevated pollinator and natural enemy abundance and diversity [11,14]. This has the added benefit of reducing input and labor demands required for turfgrass management and can potentially augment biological control of turfgrass pests [11,15]. However, despite the demonstrated benefits to many taxa, wildflower habitats do not necessarily boost the abundance of all beneficial arthropod groups [16,17]. For example, Braman et al. [16] did not observe increased abundance of ground-dwelling spiders and ants in wildflower plots adjacent to turfgrass, representing two major natural enemy groups that are potentially overlooked by current wildflower habitat management practices. In order to fully understand how wildflower habitats support arthropod diversity, it is necessary to quantify how a broad array of taxa and functional groups respond to wildflower habitat implementation.

Although turfgrass is highly prevalent in urban areas, studies of wildflower habitats in turfgrass are relatively few compared with those in agricultural systems [11,15,16,18]. Of these studies, even fewer examine changes in arthropod taxa more than one year following habitat establishment [11,16]. Influential factors like vegetation complexity, flower abundance, and plant diversity can change with successional age in wildflower habitats [19,20]. Habitat age can consequently significantly affect the abundance and richness of several arthropod groups, with variable changes occurring over time depending on trophic level and taxon [21,22,23]. For example, Albrecht et al. [19] found that wild bee and hoverfly abundance declined two years after the establishment of wildflower strips. Predator–prey ratios have also been observed to increase in older habitats compared with newly established habitats [24]. Wildflower habitats are intended to persist for extended periods of time, requiring long-term management plans. It is therefore important to understand the multi-year effects of wildflower habitats on arthropod communities in turfgrass systems.

This study builds on a 2021 study by Billeisen et al. [18], which examined changes in bee abundance one year after wildflower habitat establishment in turfgrass systems. To do this, we used pan traps to continue to monitor changes in the abundance of prevalent insect taxa and functional guilds collected in turfgrass habitats one year before and two years after establishment of directly adjacent wildflower habitats. Given that wildflower plots can provide resources for diverse insect groups, we expected all examined taxa and guilds to show sustained increases in abundance post-wildflower establishment. To provide additional insight into how the trapping method may have influenced our findings, we also quantified trap color preference between groups. Ultimately, these preliminary questions will point to further research needs in a long-term wildflower habitat-establishment study.

## 2. Materials and Methods

### 2.1. Insect Sampling

Insects were collected from six managed turfgrass habitats in the Piedmont and Sandhill ecoregions of North Carolina, comprising four golf course sites and two home lawns [18] (sites summarized in Table S1, Figure S1). In 2018, insect sampling occurred on sunny days at all sites every 2–4 weeks from July to October inclusive. During each sampling bout, 15 pan traps were placed along a 68.5 m transect within the turf directly parallel to each future wildflower site, with 4.6 m between each pan trap. Pan traps were painted either blue, white, or yellow (Rust-Oleum, Vernon Hills, IL, USA), with traps alternating in color. Each pan trap was filled with approximately 120 mL of a solution of 5 mL dish detergent (Dawn, Procter & Gamble, Cincinatti, OH, USA) diluted with 3.8 L water. Pan traps were deployed between 0800–1000 h and retrieved after approximately six hrs. Collected insects were then rinsed and stored in a Whirl-Pak bag. Specimens were either washed and pinned or placed in vials with ethanol.

In October 2018, wildflower plots were cleared of small trees, shrubs, and turfgrass using a rake. The soil was then rototilled and seeded with American Meadows Southeast Pollinator Wildflower seed mix (American Meadows, Shelburne, VT, plant species listed in Table S2) at a density of 22.68 kg seed/acre according to the seed mix instructions. This mix contained 17 plant species, including species that are native and non-native to the southeastern USA. Surveyors noted any species that successfully flowered within the plots, as well as any non-planted species that flowered. In 2019, surveyors sampled insects at all sites every 2–4 weeks from May to October using the methods described above. In October 2019, sites were mowed and re-seeded. In early 2020, large trees and shrubs were removed from the sites. Surveyors resumed sampling at all sites every 2–4 weeks from July to October 2020. Pesticide inputs were minimal at golf course sites in early 2020 due to reduced management as a result of the COVID-19 pandemic.

### 2.2. Identification

All insects were identified to family, superfamily (for Cynipoidea, Chalcidoidea, and Proctotrupoidea), or order (Thysanoptera and a single damaged Zygopteran specimen) (henceforth “families”) [25,26,27]. All relevant families were assigned to four functional groups of conservation concern: bees, butterflies, predatory flies, and wasps. We examined the response of these taxa as collective functional groups or feeding guilds (henceforth “guilds”), due to their high abundance in our sampling and their relevance as beneficial taxa in turfgrass systems. Bees and butterflies were assigned to separate guilds due to differences in feeding strategy through development, which may have affected their responses to wildflower plantings. Similarly, flies and wasps were examined as separate predatory groups to determine whether these guilds had different responses to these habitats. “Predatory” in this instance refers to families with both predatory and parasitoid feeding strategies. Though we recognize that these four guilds contain families with diverse life-history strategies, we elected to focus on the dominant reported feeding strategy in an effort to represent broad responses across guilds. For bees specifically, our collections overwhelmingly comprised non-brood parasites, which further supported treating bees as a distinct guild.

### 2.3. Data Analysis

To standardize the sampling period across years, we excluded from analyses samples that were collected in May and June, since these months were not sampled in 2018 and 2020. For every unique combination of site and date, we pooled family and guild abundance values across all traps. We analyzed families and guilds with at least 30 individuals pooled across all years and for which model conversion was feasible. To determine whether the abundance of each insect family or guild captured per sampling event differed within and across years, we constructed separate generalized linear mixed models for each family and guild using a negative binomial distribution. We elected to use this distribution to account for overdispersion caused by an excess number of zeros in the data. In each model, the year, the Julian day, and their interaction were included in the model as explanatory variables, with Julian day included to account for the variable dates of sampling bouts. Year was treated as a factor and Julian day was treated as a continuous variable. Site identity was included in the models as a random effect. Site type (golf versus home lawn) and size were not included as factors because these were not found to have an effect on bee abundance in the study by Billeisen et al. [18].

To quantify trap color preferences between guilds, we pooled the abundance of each guild across all dates for each combination of trap color and site. We then ran a linear mixed-effects model for each guild, wherein abundance was the response variable, color was the explanatory variable, and site was included as a source of random variation. A constant of 1 was added to the abundance, which was then square-root transformed to improved normality.

Statistical analyses were conducted using R software (v.4.2.1) [28]. Generalized linear mixed models with a negative binomial distribution were constructed using the package “glmmTMB” [29]. Linear mixed-effects models used for comparing trap bias were constructed using the package ‘lme4’ [30]. All models were tested using the ‘Anova’ function within the package ‘car’ [31]. Where interactions were significant, type III sums of squares were calculated. For families and guilds with a significant main effect of year and no interaction effect, we compared pairwise year means using the Tukey method in the package ‘emmeans’ [32]. Sampling abundance curves were generated using the package ‘BiodiversityR’ [33].

## 3. Results

The number of blooming plant species at each site varied from 2–16 species, including both seed-mix plants and non-planted species (summarized in Table S2). Out of the 17 seed-mix species, the percentage of species that successfully bloomed varied from 12% to 65% between sites. There were two plant species, *Aster novae-angliae* and *Lobularia maritima*, that did not successfully flower at any site. In total, we collected 8172 insects across three years of sampling, comprising eight orders and 64 families (Table S3). Of this total number, 3762 (44.1%) were in Dolichopodidae, 2197 (26.8%) were in Sarcophagidae, and 512 (6.3%) were in Halictidae. We collected 35 unique insect families in 2018, 54 in 2019, and 37 in 2020. A sampling abundance accumulation curve of family richness indicated that family richness in 2019 and 2020 was similar. The overall family diversity in 2018 did not approach an asymptote, suggesting that sampling may have inadequately captured family diversity during that year (Figure S2). Abundance of insects per sampling date varied widely, with a range of 1–131 individuals captured on a given sampling date pooled across all sites and pan traps (Figure S3).

### 3.1. Family Abundance

**Figure 1:**
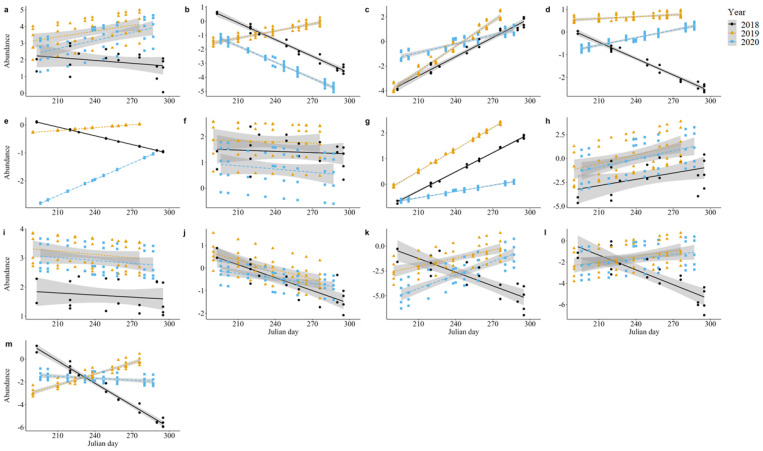
Model estimates of average number of individuals for each family per sampling bout by Julian day: (**a**) Dolichopodidae, (**b**) Formicidae, (**c**) Syrphidae, (**d**) Calliphoridae, (**e**) Cicadellidae, (**f**) Halictidae, (**g**) Hesperiidae, (**h**) Ichneumonidae, (**i**) Sarcophagidae, (**j**) Apidae, (**k**) Chloropidae, (**l**) Crabronidae, and (**m**) Miridae. Shaded bars indicate confidence intervals. Black circles indicate 2018 estimates, orange triangles indicate 2019 estimates, and blue squares indicate 2020 estimates.

Results of models comparing abundance of families and guilds over time are summarized in Table 1. Of the 13 insect families included in our analyses, 3 exhibited a significant interaction effect between year and Julian day on abundance per sampling event. These included Dolichopodidae (Χ^2^ = 10.41, *p* < 0.01, Figure 1a), Formicidae (Χ^2^ = 9.41, *p* < 0.01, Figure 1b), and Syrphidae (Χ^2^ = 11.15, *p* < 0.01, Figure 1c). There were generally more dolichopodids in late 2019 than late 2018 (Figure 1a). There were more syrphids in early 2020 compared with early 2018 and early 2019 (Figure 1c). Formicids and dolichopodids showed an apparent drop in abundance between equivalent periods in subsequent years, with more individuals in late 2019 compared with late 2020 (Figure 1b).

We observed a significant year effect for six families: Calliphoridae (Χ^2^ = 6.63, *p* = 0.04, Figure 1d), Cicadellidae (Χ^2^ = 11.26, *p* < 0.01, Figure 1e), Halictidae (Χ^2^ = 16.43, *p* < 0.01, Figure 1f), Hesperiidae (Χ^2^ = 33.61, *p* < 0.01, Figure 1g), Ichneumonidae (Χ^2^ = 29.68, *p* < 0.01, Figure 1h), and Sarcophagidae (Χ^2^ = 23.93, *p* < 0.01, Figure 1i). There were significantly more ichneumonids and sarcophagids in 2019 (ichneumonids: z = 4.01, *p* < 0.001; sarcophagids: z = 4.56, *p* < 0.001) and 2020 (ichneumonids: z = 2.98, *p* < 0.01; sarcophagids: z = 4.27, *p* < 0.001) compared with 2018. There were also significantly more calliphorids in 2019 than 2018 (z = 2.43, *p* = 0.04). Several groups showed an apparent drop in abundance between subsequent years, with significantly more cicadellids (z = 3.64, *p* < 0.001), halictids (z = 4.06, *p* < 0.001), and hesperiids (z = 5.56, *p* < 0.001) in 2019 than in 2020. There were also more cicadellids in 2018 than in 2020 (z = 2.64, *p* = 0.02).

There was a significant effect of Julian day on abundance per sampling bout for Apidae (Χ^2^ = 6.24, *p* = 0.01, Figure 1j), Hesperiidae (Χ^2^ = 20.68, *p* < 0.001, Figure 1g), and Ichneumonidae (Χ^2^ = 25.93, *p* < 0.001, Figure 1h), with a slight increase in abundance over a given year for all families.

There was no significant effect of either year, Julian day, or their interaction on the abundance of Chloropidae, Crabronidae, or Miridae (0.05 < *p* < 0.83, Figure 1k–m).

### 3.2. Guild Abundance

Significant majorities of butterflies and bees were each represented by a single family (bees: 77% Halictidae; butterflies: 93% Hesperiidae). The results of these analyses closely resembled those of the analyses for the dominating family. Therefore, we focused on the analyses of the two predatory groups: flies and wasps.

**Figure 2:**
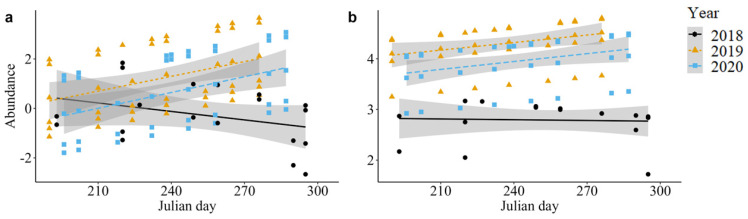
Model estimates of average number of individuals for each guild per sampling bout by Julian day: (**a**) wasps, and (**b**) predatory flies. Shaded bars indicate confidence intervals. Black circles indicate 2018 estimates, orange triangles indicate 2019 estimates, and blue squares indicate 2020 estimates.

Wasps exhibited a significant interaction effect between year and Julian day on abundance per sampling event (Χ^2^ = 12.22, *p* < 0.01, Figure 2a), with a general higher abundance of wasps in late 2019 compared with late 2018.

There was a significant effect of year on predatory fly abundance (Χ^2^ = 59.48, *p* < 0.001, Figure 2b), with significantly fewer individuals per bout in 2018 than in 2019 (z = −7.06, *p* < 0.0001) or 2020 (z = 5.41, *p* < 0.0001). Neither Julian day (Χ^2^ = 1.74, *p* = 0.19) nor the interaction between Julian day and year (Χ^2^ = 3.00, *p* = 0.22) had a significant effect on predatory fly abundance.

### 3.3. Trap Color Preference

**Figure 3:**
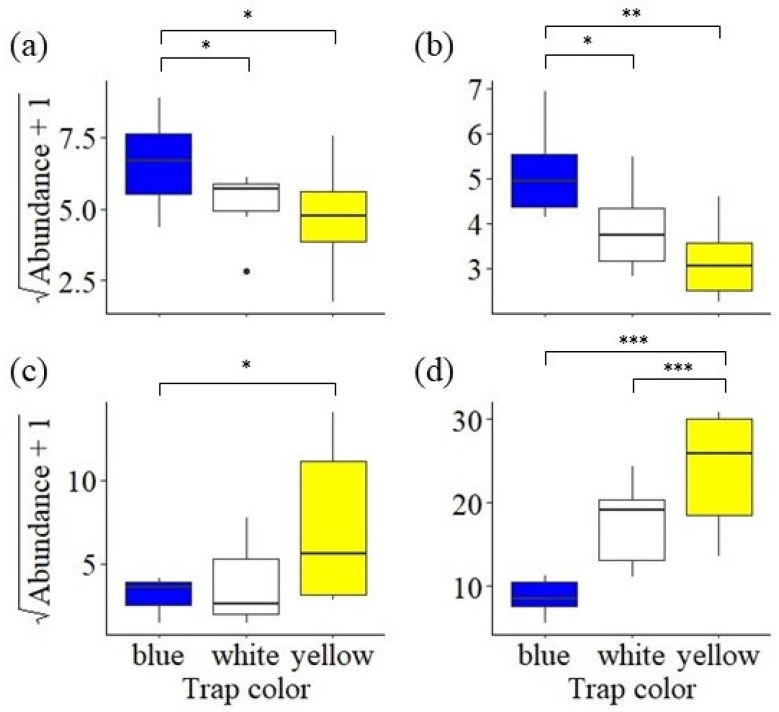
Abundance of guilds per site grouped by date and trap color: (**a**) bees, (**b**) butterflies, (**c**) wasps, and (**d**) predatory flies (* *p* < 0.05, ** *p* < 0.01, *** *p* < 0.0001).

There was a significant effect of trap color on abundance of all examined guilds (bees: F_2,10_ = 7.05, *p* = 0.01, Figure 3a; butterflies: F_2,10_ = 7.05, *p* = 0.002, Figure 3b; wasps: F_2,10_ = 41.54, *p* < 0.001, Figure 3c; predatory flies: F_2,7.2_ = 8.27, *p* = 0.01, Figure 3d). Note that, as stated above, the bees largely comprised halictids and the butterflies largely comprised hesperiids. Bee abundance was significantly higher in blue traps compared with both white traps (t_10_ = 2.74, *p* < 0.05) and yellow traps (t_10_ = 3.59, *p* = 0.01). Similarly, butterfly abundance was also significantly higher in blue traps compared with white traps (t_10_ = 3.24, *p* = 0.02) and yellow traps (t_10_ = 5.06, *p* = 0.001).

Wasps and predatory flies showed a somewhat reversed preference pattern compared with bees and butterflies. More wasps were caught in yellow traps than in blue traps (t_7.4_ = 3.71, *p* = 0.02). Wasps were also more abundant by a marginally non-significant amount in yellow traps compared with white traps (t_7.0_ = 2.93, *p* = 0.05). Predatory fly abundance was significantly higher in yellow traps compared with both white traps (t_10_ = 3.81, *p* = 0.009) and blue traps (t_10_ = 9.08, *p* < 0.001).

## 4. Discussion

As predicted, none of the examined groups decreased in overall abundance from 2018 (pre-wildflower implementation) to directly post-implementation in 2019. However, several families, including Apidae, Chloropidae, Crabronidae, and Miridae, showed no change in abundance between years, indicating no significant effect of wildflower habitat. This trend was particularly unexpected concerning apids, since bees typically depend on floral resources for at least part of their life cycle.

Some families (Cicadellidae, Halictidae, and Hesperiidae) showed significant declines in abundance in 2020 compared with 2019. Certain families (Formicidae and Dolichopidae) were also less abundant in late 2020 compared with similar time periods in previous years. The reasons for these declines are not immediately clear but may have been due to several interacting factors. First, we anecdotally observed a decline in flower abundance at our sites in 2020. Out of the 16 plant species included in the American Meadows Southeast Pollinator Wildflower seed mix, 9 are annual, and the self-seeding success of these species is not known. In agricultural systems, wildflower strips tend to have more perennial plant species and lower plant diversity as succession progresses [34]. Although the sites were re-seeded in 2019, the seeds were not placed on bare soil as they had been in 2018, which may have reduced germination success. In addition, although trees and large shrubs were removed in early 2020, other small plants were not removed except for mowing. This may have contributed to an overall decline in floral abundance in our plots. Reduced floral resources may have played a role in the reduced numbers of halictids and hesperiids in 2020, as abundance of bees and butterflies can respond strongly to variations in floral resource abundance [35,36,37]. These results point to the necessity of continued active management of wildflower habitats, as well as that of maintaining mixed successional-stage wildflower habitats to support a large diversity of arthropod taxa [38]. Interguild interactions were another factor in this system that may have affected abundance between years. The effects and repercussions of interguild predation are variable and often difficult to predict [39,40]. For example, overall predatory fly abundance was higher in 2019 and 2020 than 2018, while wasps tended to be frequent in both late 2019 and late 2020. This surge in predators and parasitoids may have potentially depressed prey groups.

Another factor that may have contributed to these declines was staggered pesticide input in 2020. Early in the 2020 growing season, land managers at golf courses were unable to make regular pesticide applications due to the COVID-19 pandemic. Therefore, individuals that were released from pesticide pressure early in the year may have been subsequently hit during a vulnerable life-history stage when management practices resumed later in the year. Both hesperiids and apids showed slight but significant increases in abundance as the year progressed, and thus were potentially especially affected by latent inputs in 2020. The most common butterfly species in our samples were *Hylephila phyleus* (Drury, 1973) and *Lerema accius* (J.E. Smith 1797), both of which depend on grasses as larval host plants [41,42] and would therefore be affected directly by turf pesticides. Reduced chemical inputs in 2020 may have also caused an increase in arthropod prey that was otherwise not well detected in our pan traps, thereby boosting the populations of predator groups. However, it remains unclear to what degree chemical inputs played a role in these declines. To disentangle these effects, it may be necessary to monitor arthropod populations in turfgrass systems with variable pesticide inputs.

The abundance and diversity of flowering plant species probably also played a role in the patterns we observed. Though we did not record percent cover, we observed a wide range of blooming plant diversity between our sites. The diversity and display size of flowering plants strongly influence the abundance and diversity of visiting arthropod species [43,44,45]. None of the sites achieved the full diversity of successfully flowering species, which is typical of previous studies of seed mixes [45]. The identity and diversity of wildflower mixes and their abilities to attract and support various insect groups is an area of active study. However, much of the existing research focuses on agricultural areas, and the ideal wildflower mixes for turfgrass systems remain unclear.

Our study does not account for factors related to weather. It is well established that climatic variation, including precipitation and average temperature, can also strongly influence arthropod abundance. This includes direct impacts via altered development times and survival, as well as indirectly via changes in floral resources [46,47]. However, this variation further rationalizes the need for more multi-year studies on the influences of wildflower habitats on arthropod communities. Data from long-term datasets can serve to disentangle the effects of site management and climatic variability on the success of these communities. In addition to observing variable abundance across years, we also observed variable efficiency between trap colors. Pan trapping is low-cost and requires little training, making it an ideal method for long-term monitoring studies with limited staff and time. Given the popularity of using pan traps for monitoring arthropod populations, an increasing number of studies have explored how color, timing, and context influence results [48,49]. We observed a bias for blue in bees, which aligns with findings from some previous studies [50,51] but differs from others [52,53]. We also observed a similar bias for blue in butterflies. This is of note because the pan trap method is not frequently used for lepidopterans, given the risk of damaging specimens. However, we observed large numbers of butterflies of the family Hesperiidae in our pan traps, perhaps pointing to the potential use of pan traps for sampling this group. Conversely to the bees and butterflies, our predatory groups—flies and wasps—exhibited a preference for yellow. Wasps and syrphid flies have previously been observed to have a bias for yellow [49,54,55,56]. The flipped preference patterns of pollinators and natural enemies in this study point to the value in using multiple trap colors when sampling for beneficial insects in turfgrass systems.

It is worth noting that pan traps may be inadequate for estimating the abundance of certain taxa. Sticky traps are known to be an effective method for sampling parasitoid wasps [57]. Therefore, overall parasitoid wasp abundance may be poorly reflected by our study. In addition, we did not employ pitfall traps, which are a common method for trapping ground-crawling predators such as carabids and spiders [16,58]. Other studies have observed changes in ground-crawling predator abundance between years in turfgrass systems when using pitfall traps [17,59]. Ground-crawling predators therefore represent another major component of the natural enemy community which is likely to have been overlooked in the present study.

This study points to a number of research priorities for turfgrass systems. Long-term arthropod monitoring beyond the scope of this study (i.e., >2 years post-wildflower establishment) is essential. Using a long-term wildflower plot, researchers can track diverse arthropod taxa over multiple years, tease out intraguild interactions, and monitor how turfgrass management practices may affect arthropod diversity and abundance. Ultimately, the goal of a long-term monitoring project would be to identify best practices for maintaining the benefits of wildflower habitats over many years. Between 2005 and 2017, approximately 46% of golf courses expanded the amount of natural habitat present on the course [60], pointing to the increasing need for research-based management. This rise also points to widespread trends within the industry towards reducing areas of intensively managed turfgrass, and the opportunities for creating more impactful conservation areas are substantial. In the southeastern USA, urban and suburban areas are expected to increase by 200–300% by 2060 [61]. Given the increased predominance of urban systems, it is essential to tailor management of wildflower habitats in turfgrass systems in a way that mitigates the negative effects of urbanization on arthropods.

## Figures and Tables

**Table 1:** Results of general linear mixed model examining abundance of each family and guild per sampling event (ANOVA (type II or III Wald Chi-square test)). Starred values indicate significance (α = 0.05).

Grouping		Year			Julian Day			Year * Julian Day		
	Group	Χ^2^ Statistic	df	*p*-Value	Χ^2^ Statistic	df	*p*-Value	Χ^2^ Statistic	df	*p*-Value
Family	Apidae	0.44	2	0.80	6.24	1	0.01 *	0.71	2	0.70
	Calliphoridae	6.63	2	0.04 *	0.00	1	0.95	1.55	2	0.46
	Chloropidae	4.68	2	0.10	1.92	1	0.17	4.59	2	0.10
	Cicadellidae	11.26	2	<0.01 **	0.14	1	0.71	3.08	2	0.21
	Crabronidae	0.38	2	0.83	1.30	1	0.25	3.86	2	0.14
	Dolichopodidae	6.18	2	<0.05 *	2.73	1	0.10	10.41	2	<0.01 **
	Formicidae	8.40	2	0.02 *	3.76	1	0.05	9.41	2	<0.01 **
	Halictidae	16.43	2	<0.001 ***	2.59	1	0.11	1.22	2	0.54
	Hesperiidae	33.61	2	<0.001 ***	20.68	1	<0.001 ***	4.52	2	0.10
	Ichneumonidae	29.68	2	<0.001 ***	25.93	1	<0.001 ***	1.06	2	0.69
	Miridae	5.65	2	0.06	2.41	1	0.12	5.87	2	0.05
	Sarcophagidae	23.93	2	<0.001 ***	1.26	1	0.26	0.02	2	0.99
	Syrphidae	7.35	2	0.03 *	40.98	1	<0.001 ***	11.15	2	<0.01 **
Guild	Wasps	8.62	2	0.01 *	4.11	1	0.04	12.22	2	<0.01 **
	Predatory flies	59.48	2	<0.001 ***	1.74	1	0.19	3.00	2	0.22

Significance levels: * *p* < 0.05, ** *p* < 0.01, *** *p* < 0.001.

## Data Availability

The data presented in this study and the code used for analysis are openly available in the GitHub repository ‘https://github.com/lhamon31/Hamon.et.al.2023.TurfgrassPollinators (accessed on 1 June 2024)’.

## Supplementary Materials

The following supporting information can be downloaded at: https://www.mdpi.com/article/10.3390/insects15070520/s1, Table S1: Study site locations and their distance from North Carolina State University campus.; Table S2: Seed-mix components and other flowers that flowered in each site in 2019–2020. The seed mix was the American Meadows Southeast Pollinator Wildflower Seed Mix^®^. Table S3: Specimen counts and guild assignments by family, superfamily, or order; Figure S1: Aerial images of the approximate extent of wildflower plots; Figure S2: Family accumulation curve with 95% confidence interval against number of sampling events; Figure S3: Total abundance of collected insects pooled by sampling date.
