# Supplementary material for: The Impact of Wildflower Habitat on Insect Functional Group Abundance in Turfgrass Systems
Source: Insects. 2024 Jul 11;15(7):520. doi: 10.3390/insects15070520 (PMC11277235; doi:10.3390/insects15070520)
Supplement: Supplementary file 1 [file insects-15-00520-s001.zip › Supplemental Figure S1.pdf]

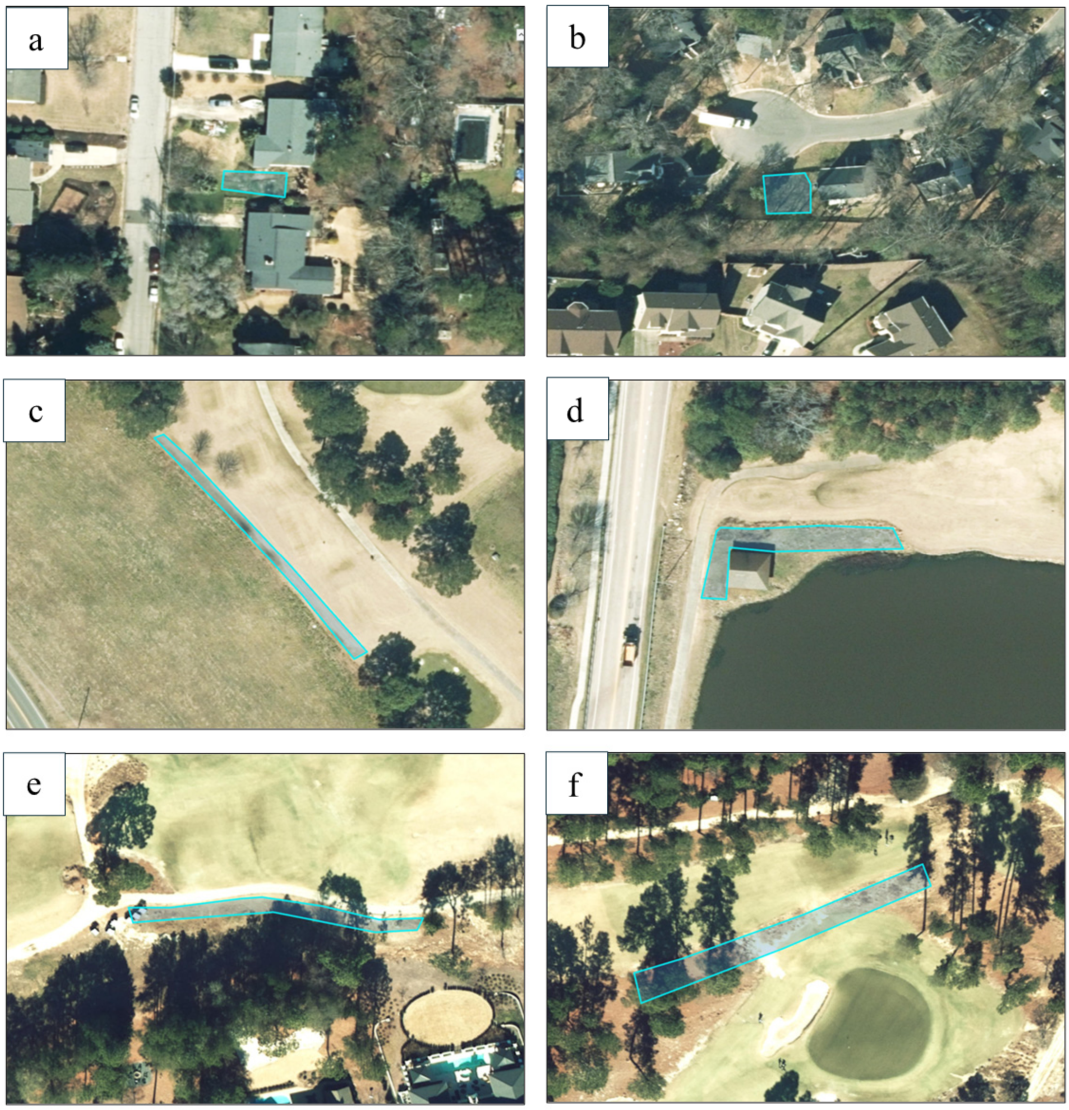


**Supplemental Figure S1.** Aerial images of the approximate extent of wildflower plots. (a) HL2 and (b) HL3 are home lawn sites, while (c) GCL3, (d) GCH5, (e) GCH6 and (f) GCH7 are golf course sites. Aerial images sources: NC CGIA, Maxar, Microsoft.
