# Supplementary material for: The Impact of Wildflower Habitat on Insect Functional Group Abundance in Turfgrass Systems
Source: Insects. 2024 Jul 11;15(7):520. doi: 10.3390/insects15070520 (PMC11277235; doi:10.3390/insects15070520)
Supplement: Supplementary file 1 [file insects-15-00520-s001.zip › Supplemental Figure S2.pdf]

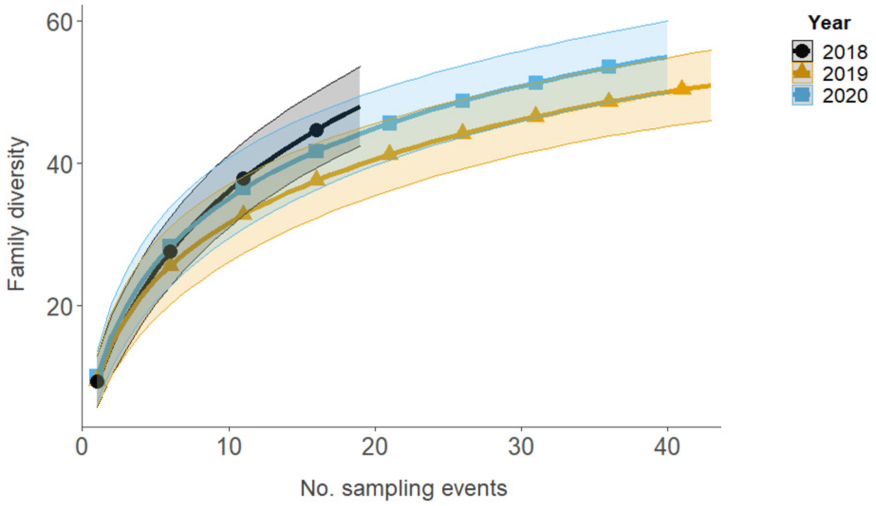

Supplemental Figure S2. Family accumulation curve with 95% confidence interval against number of sampling events. Black circles indicate 2018 estimates, orange triangles indicate 2019 estimates, and blue squares indicate 2020 estimates.
