# Supplementary material for: The Impact of Wildflower Habitat on Insect Functional Group Abundance in Turfgrass Systems
Source: Insects. 2024 Jul 11;15(7):520. doi: 10.3390/insects15070520 (PMC11277235; doi:10.3390/insects15070520)
Supplement: Supplementary file 1 [file insects-15-00520-s001.zip › Supplemental Table S1.pdf]

Supplemental Table S1. Study site locations and their distance from North Carolina State University campus.

Site	Type	Coordinates	County	Distance from Campus (km)
HL2	Home lawn	(35.7759, -78.7046)	Wake	3.20
HL3	Home lawn	(35.8771, -78.6695)	Wake	10.0
GCL3	Golf Course	(35.6251, -78.7758)	Wake	20.3
GCH5	Golf Course	(35.9505, -78.5004)	Wake	23.9
GCH6	Golf Course	(35.1963, -79.4590)	Moore	96.9
GCH7	Golf Course	(35.1984, -79.4548)	Moore	96.5
