# Supplementary material for: The Impact of Wildflower Habitat on Insect Functional Group Abundance in Turfgrass Systems
Source: Insects. 2024 Jul 11;15(7):520. doi: 10.3390/insects15070520 (PMC11277235; doi:10.3390/insects15070520)
Supplement: Supplementary file 1 [file insects-15-00520-s001.zip › Supplemental Table S2.pdf]

Supplemental Table S2. Seed mix components and other flowers that flowered in each site in 2019-2020. The seed mix was the American Meadows Southeast Pollinator Wildflower Seed Mix®. "X" indicates that this flower was observed blooming in this site. Flowering information was not recorded for GCH7

	Common Name	Scientific Name	HL2	HL3	GCL3	GCH5	GCH6
**American Meadows Southeast Pollinator Wildflower Seed Mix® (AM)**	New England aster	*Aster novae-angliae*					
	Lance-leaf coreopsis	*Coreopsis lanceolata*					X
	Plains coreopsis	*Coreopsis tinctoria*	X		X	X	X
	Sulphur cosmos	*Cosmos sulphureus*		X	X	X	
	Purple coneflower	*Echinacea purpurea*					X
	Blanket flower	*Gaillardia pulchella*	X	X	X	X	X
	Rose mallow	*Lavatera trimestris*	X	X	X		
	Four o'clock	*Mirabilis jalapa*		X			
	Clasping coneflower	*Rudbeckia amplexicaulis*	X			X	X
	Black-eyed Susan	*Rudbeckia hirta*	X		X	X	X
	Scarlet sage	*Salvia coccinea*		X	X	X	X
	Butterfly weed	*Asclepias tuberosa*	X				X
	Siberian wallflower	*Cheiranthus allionii*					X
	Wild cosmos sensation mix	*Cosmos bipinnatus*	X				
	Chinese forget me not	*Cynoglossum amabile*		X	X	X	X
	Sweet alyssum	*Lobularia maritima*					
	Red poppy	*Papaver rhoeas*	X			X	X
**Not in mix**	Common Spiderwort	*Tradescantia virginiana*					X
	Venus' looking-glass	*Triodanis perfoliata* subsp. *biflora*			X	X	X
	Beebalm	*Monarda didyma*					X
	Partridge pea	*Chamaecrista fasciculata*					X
	Common mugwort	*Artemisia vulgaris*				X	
	Sweet William catchfly	*Silene armeria*				X	
	Passionflower	*Passiflora* sp.				X	
	Cornflower	*Centaurea cyanus*	X		X		
	Pineweed	*Hypericum gentianoides*					X
