# Supplementary material for: The Impact of Wildflower Habitat on Insect Functional Group Abundance in Turfgrass Systems
Source: Insects. 2024 Jul 11;15(7):520. doi: 10.3390/insects15070520 (PMC11277235; doi:10.3390/insects15070520)
Supplement: Supplementary file 1 [file insects-15-00520-s001.zip › Supplemental Table S3.pdf]

Supplemental Table S3. Specimen counts and guild assignments by family, superfamily, or Order.

Order	Taxon	Guild assignment	Number of individuals
Coleoptera	Cantharidae	n/a	3
	Chrysomelidae	n/a	26
	Curculionidae	n/a	4
	Kateretidae	n/a	1
	Mordellidae	n/a	9
	Nitidulidae	n/a	5
	Orsodacnidae	n/a	1
	Scarabaeidae	n/a	4
Diptera	Anthomyiidae	n/a	10
	Calliphoridae	n/a	50
	Canacidae	n/a	1
	Cecidomyiidae	n/a	7
	Chironomidae	n/a	1
	Chloropidae	n/a	38
	Drosophilidae	n/a	4
	Empididae	n/a	15
	Lonchaeidae	n/a	2
	Muscidae	n/a	132
	Phoridae	n/a	4
	Sciaridae	n/a	6
	Stratiomyidae	n/a	3
	Tephritidae	n/a	1
	Asilidae	predatory flies	8
	Bombyliidae	predatory flies	5
	Dolichopodidae	predatory flies	3762
	Sarcophagidae	predatory flies	2202
	Syrphidae	predatory flies	196
	Tachinidae	predatory flies	13
Hemiptera	Anthocoridae	n/a	1
	Aphididae	n/a	69
	Cercopidae	n/a	21
	Cicadellidae	n/a	59
	Cydnidae	n/a	1
	Membracidae	n/a	13
	Miridae	n/a	32
	Nabidae	n/a	4
	Pentatomidae	n/a	1
	Rhyparochromidae	n/a	3
Hymenoptera	Andrenidae	bees	7
	Apidae	bees	143
	Halictidae	bees	555
	Megachilidae	bees	12
	Argidae	n/a	1
	Formicidae	n/a	35

	Braconidae	predatory wasps	9
	Chalcidoidea	predatory wasps	12
	Crabronidae	predatory wasps	47
	Cynipoidea	predatory wasps	7
	Ichneumonidae	predatory wasps	399
	Mutillidae	predatory wasps	1
	Pompilidae	predatory wasps	11
	Proctotrupoidea	predatory wasps	22
	Scelionidae	predatory wasps	3
	Scoliidae	predatory wasps	79
	Sphecidae	predatory wasps	52
	Thynnidae	predatory wasps	20
	Tiphiidae	predatory wasps	2
	Vespidae	predatory wasps	62
Lepidoptera	Hesperiidae	butterflies	308
	Lycaenidae	butterflies	2
	Nymphalidae	butterflies	12
	Papilionidae	butterflies	2
	Pieridae	butterflies	6
	Attevidae	n/a	1
	Sphingidae	n/a	1
	Zygaenidae	n/a	1
Odonata	Libellulidae	n/a	1
	Zygoptera	n/a	1
Orthoptera	Gryllotalpidae	n/a	1
Thysanoptera	Thysanoptera	n/a	3
