# Supplementary figures and images for: The Impact of Wildflower Habitat on Insect Functional Group Abundance in Turfgrass Systems
Source: Insects. 2024 Jul 11;15(7):520. doi: 10.3390/insects15070520 (PMC11277235; doi:10.3390/insects15070520)

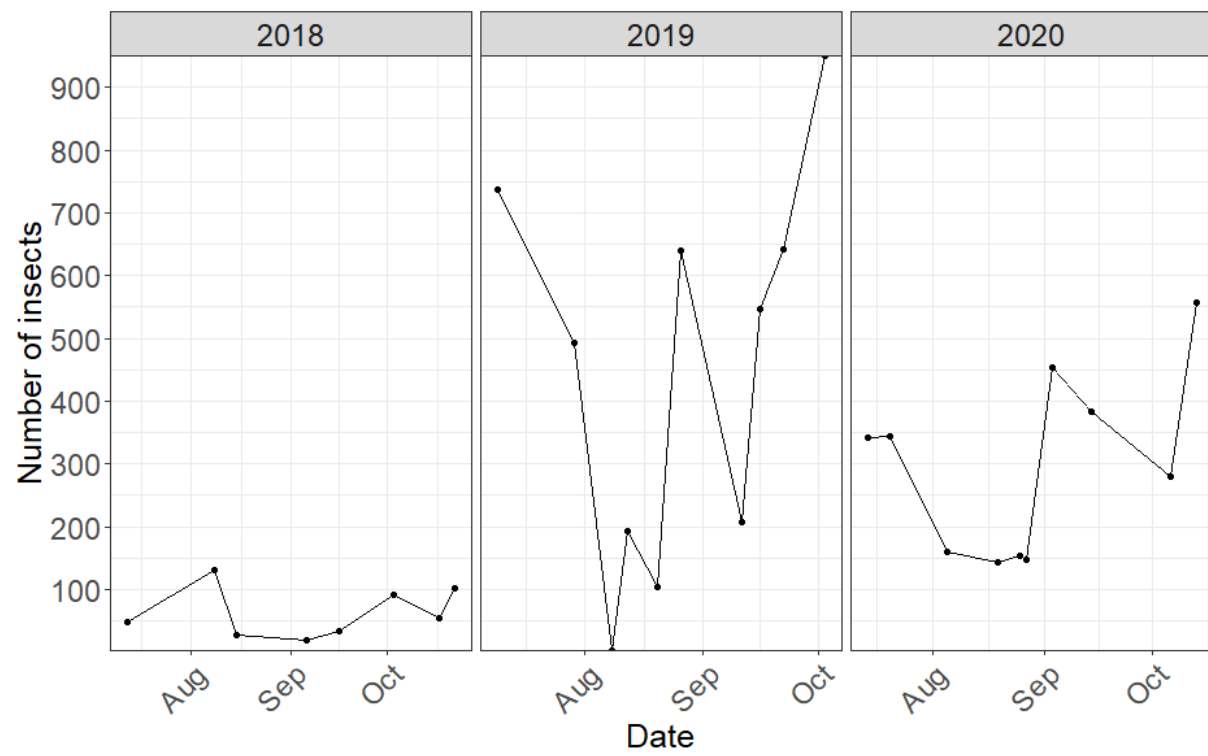

**Supplemental Figure S3.** Total abundance of collected insects pooled by sampling date.

Supplement: Supplementary file 1 [file insects-15-00520-s001.zip › Supplemental Figure S3.pdf]
